# Feasible, Efficient and Necessary, without Exception – Working with Sex Workers Interrupts HIV/STI Transmission and Brings Treatment to Many in Need

**DOI:** 10.1371/journal.pone.0121145

**Published:** 2015-10-21

**Authors:** Richard Steen, Tisha Wheeler, Marelize Gorgens, Elizabeth Mziray, Gina Dallabetta

**Affiliations:** 1 Department of Public Health, Erasmus MC, University Medical Center Rotterdam, Rotterdam, The Netherlands; 2 Office of HIV/AIDS, United States Agency for International Development, Washington, District of Columbia, United States of America; 3 The World Bank, Washington, District of Columbia, United States of America; 4 The Bill & Melinda Gates Foundation, Washington, District of Columbia, United States of America; Johns Hopkins School of Public Health, UNITED STATES

## Abstract

**Background and Overview:**

High rates of partner change in sex work—whether in professional, ‘transactional’ or other context—disproportionately drive transmission of HIV and other sexually transmitted infections. Several countries in Asia have demonstrated that reducing transmission in sex work can reverse established epidemics among sex workers, their clients and the general population. Experience and emerging research from Africa reaffirms unprotected sex work to be a key driver of sexual transmission in different contexts and regardless of stage or classification of HIV epidemic. This validation of the epidemiology behind sexual transmission carries an urgent imperative to realign prevention resources and scale up effective targeted interventions in sex work settings, and, given declining HIV resources, to do so efficiently. Eighteen articles in this issue highlight the importance and feasibility of such interventions under four themes: 1) epidemiology, data needs and modelling of sex work in generalised epidemics; 2) implementation science addressing practical aspects of intervention scale-up; 3) community mobilisation and 4) the treatment cascade for sex workers living with HIV.

**Conclusion:**

Decades of empirical evidence, extended by analyses in this collection, argue that protecting sex work is, without exception, feasible and necessary for controlling HIV/STI epidemics. In addition, the disproportionate burden of HIV borne by sex workers calls for facilitated access to ART, care and support. The imperative for Africa is rapid scale-up of targeted prevention and treatment, facilitated by policies and action to improve conditions where sex work takes place. The opportunity is a wealth of accumulated experience working with sex workers in diverse settings, which can be tapped to make up for lost time. Elsewhere, even in countries with strong interventions and services for sex workers, an emerging challenge is to find ways to sustain them in the face of declining global resources.

## Introduction

**Fig 1:**
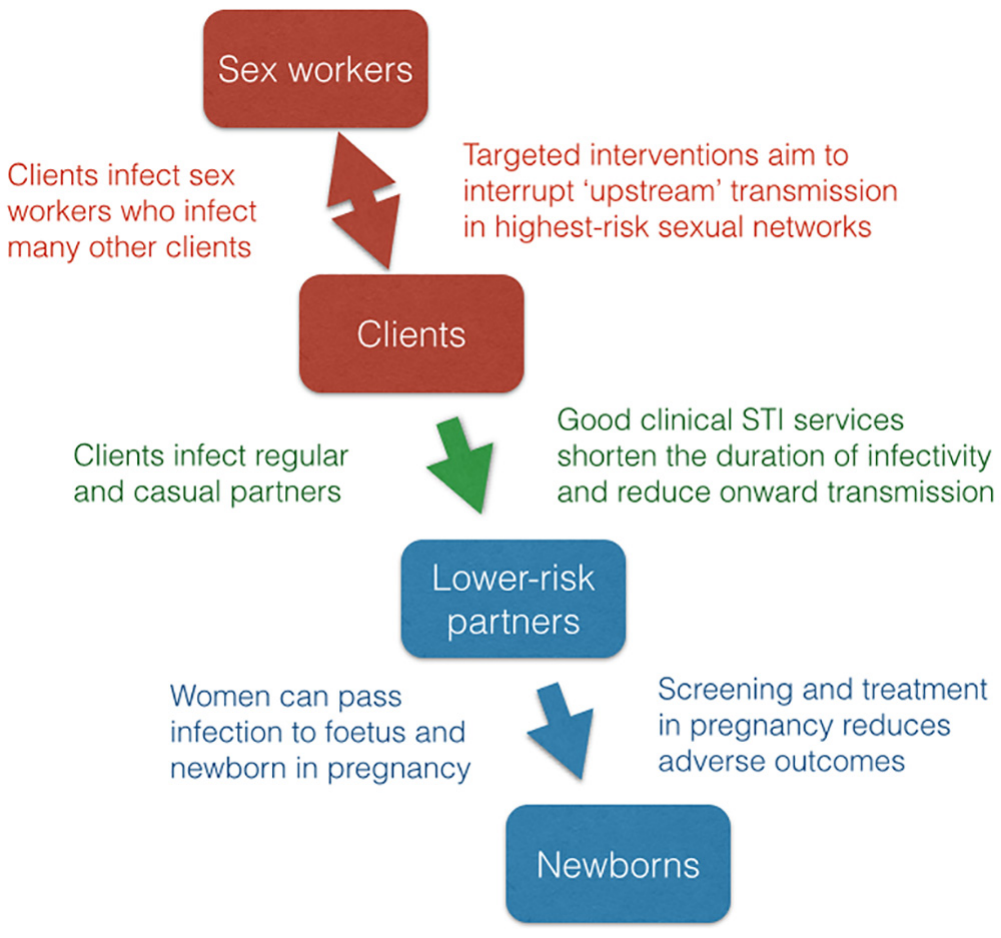
Common ‘upstream—downstream‘ transmission pathways in heterosexual networks. High-incidence transmission ‘upstream’ in sex work networks is the source of many secondary or ‘downstream’ infections among lower-risk populations.

Sex work is central to transmission of HIV and other sexually transmitted infections (STI) due primarily to the large numbers of infections that can rapidly follow each new transmission event. When condoms are used inconsistently, clients quickly infect sex workers who bear the heaviest burden of disease and complications.[1,2] High rates of partner change in sex work facilitate not only rapid acquisition, but also onward transmission to other clients, seeding multiple new streams of infection.[3,4] Clients in turn infect other sexual partners—sex workers as well as regular and casual partners—creating waves of secondary transmission that ripple outward at lower incidence rates for years. Such transmission dynamics, not merely higher prevalence, underlie the importance of sex work to HIV transmission. The flow of new infections generated efficiently ‘upstream’ becomes the source of flooding ‘downstream’ in the general population (Fig 1).

Fortunately, high-incidence transmission in sex work can be interrupted. Several countries have demonstrated the feasibility of protecting female sex workers and their clients from HIV/STI transmission, and averting subsequent secondary or ‘downstream’ transmission in the wider population. When interventions are implemented at sufficient scale, as in Thailand, Cambodia and parts of India, for example, established HIV/STI epidemics have been halted and reversed.[5–8] The cost-effectiveness of such interventions has been estimated to be high, even cost-saving when ART is factored in.[9–11] Numerous studies from Africa have replicated findings of feasibility and effectiveness.[12–15] Recent research from the continent repositions sex work—when not protected by consistent condom use—as an important epidemic driver.[16–19]

Sex work often overlaps with drug-injecting populations, men who have sex with men and transgender persons, expanding transmission potential beyond heterosexual networks. Heterogeneity of sexual behaviour and conditions under which sex work takes place influence to a great extent the degree of vulnerability and risk in sex work, the potential for transmission within larger epidemics, as well as intervention options.[20,21] For example, ‘transactional sex’, occasional or part-time sex work may be more common than overt sex work in some settings, but may also be less accessible to programmes and, if client numbers are small, less important in overall transmission.

This PLoS library collection of eighteen articles extends our understanding of the dynamics of sex work, the potential impact of interventions, and the implementation science behind successful programmes. New research and modelling refine concepts, analytical methods and estimates of the many secondary infections attributable to sex work in generalised HIV epidemics. Other articles, focusing mainly on female sex workers, add to an extensive body of empirical experience which already exists to inform programmes, addressing questions and challenges of sustainability, cost effectiveness, scale and quality.[22–31]

### Understanding sex work and HIV transmission

Despite an early focus on preventing HIV/STI transmission in sex work, few African countries currently implement interventions in sex work settings to scale.[13,32–37] Sex work occupies a marginal place in national plans, if at all, and resource allocation for targeted interventions is typically small.[38] This under-allocation can be partly explained by common misconceptions about sex work networks, which underestimate their role in generalised HIV epidemics, by methods used to identify epidemic drivers and set prevention priorities, and by data gaps.[16–19]

Mishra et al. rigourously deconstruct the logic and evidence behind such assumptions in two papers beginning with a systematic review of empirical and model-based evidence on the role of sex work in HIV transmission.[39] Drawing on over 200 studies, the authors examine the rationale for focusing on sex work and describe limitations of commonly used methods for estimating the contribution of sex work to HIV epidemics. The modes of transmission (MOT) and classic population attributable fraction (PAF) measures—estimated for half to two-thirds of generalised African HIV epidemics—attribute only 2–7% of new infections to sex work, and underestimate its medium to long-term contribution to overall HIV transmission. Two important reasons for this are underestimating population sizes—particularly of clients based on data from household surveys—and failing to factor in onward or secondary transmission arising from sex work. Using dynamic HIV models with data from Benin, Burkina Faso and Kenya, the authors provide alternative estimates, ranging from 14–38% in areas with moderate to high condom use, to 60–88% in sex work settings unreached by interventions. They conclude that focusing HIV prevention programmes on sex workers and clients remains critical to the HIV response across sub-Saharan Africa, even in countries with large generalised HIV epidemics.

The second paper by Mishra et al. critically examines the MOT methods that are widely used to set intervention priorities in different types of epidemics.[40] The validation was done by first creating synthetic models of concentrated, mixed and generalised epidemics, using data from India, Kenya and Lesotho, then performing MOT analyses on each epidemic scenario. In this way, the MOT metric was examined, in simple form and adjusting for potential biases and limitations, and its utility in identifying epidemic drivers evaluated. The analysis identified three important problems: 1) biases and structural limitations of the MOT metric itself, 2) failure to identify relevant prevention targets over time, and 3) misleading estimates of the contribution of different subgroups to overall transmission in the short and long term. The implications for prevention, modelled separately and assuming a fixed resource cap, are substantial. Using the generic MOT diverted resources away from important epidemic drivers and resulted in increased overall HIV incidence, while factoring in longer-term transmission (long-term PAF_t_) achieved the largest long-term impact. The authors conclude that the MOT is not a valid tool to identify epidemic drivers and is of limited value in guiding HIV prevention priorities. Other analyses have identified similar limitations, while improved methods in Nigeria resulted in considerably higher estimates of transmission attributable to sex work.[18, 19]

A concrete example of MOT-directed policies can be seen in Swaziland. With one of the highest national HIV prevalence rates in the world, virtually all prevention efforts—behaviour change, male circumcision and ART for its prevention effects—are spread widely across the general population. Yet, Baral et al. surveyed over three-hundred young, active, full-time sex workers who were easy to identify, had HIV prevalence in excess of 70% and reported large sexual networks and low levels of condom use.[41] They conclude that these factors likely result in high per-act HIV transmission rates that contribute significantly to Swaziland's epidemic. Similarly, in Mozambique, with about 11% adult HIV prevalence, interventions for sex workers were only recently included in national plans. Langa et al. describe interviews with over two hundred independent, street-based female sex workers who reported high numbers of clients, inconsistent condom use and general distrust of health services.[42]

In contrast, Benin is one of the few African countries that has implemented sex work interventions to scale. Williams et al. used extensive epidemiological and intervention data to parameterise a model which estimates that 96% of HIV infections before the start of interventions were linked directly or indirectly to sex work.[43] Over the next 15 years, observed increases in condom use likely prevented nearly two-thirds of new HIV infections among female sex workers and one-third among the general population.

Despite the importance of sex work to HIV/STI transmission, resources available to support targeted interventions have been estimated to be less than one percent of programme expenditures in countries with generalised HIV epidemics. [44] Better alignment of policies, programming and resource allocation is clearly needed to strengthen sex work programming in such settings.

### Implementing to scale for impact

The actual scale of the response—targeted geographic coverage with adequate intensity and sustainability of cost-effective services—are arguably critical factors that determine the course of epidemics. Notably in Asia, large-scale implementation of basic interventions in sex work settings, supported by structural change, has turned around several rapidly expanding HIV epidemics.[6–8] Our understanding of the important parameters and approaches to achieving scale efficiently—standardised intervention packages implemented by all service providers in the right areas, capacity building, micro-planning, monitoring for management, etc.–have advanced considerably with recent experience.[20,31] A systematic review of sex work interventions in Africa since 2000 found sufficient evidence from 26 studies to inform delivery of peer interventions, condom promotion and STI services that directly reduce HIV exposure and transmission efficiency among sex workers.[13] Few prevention and treatment programmes, however, were implemented at sufficient scale and intensity, and most did little to improve structural conditions of sex work. Several papers in this collection describe the implementation science behind targeted interventions in sex work settings—how to plan for scale, implement efficiently and monitor progress.

Two papers report on national-level mapping exercises conducted in Africa using methods—including confidentiality safeguards—adapted from Indian experience. In Kenya, interviews with secondary key informants were used by Odek et al. to identify ‘hot spots’, characterise how sex work takes place and estimate numbers of female sex workers.[14] Population estimates also provide data for planning, implementing and monitoring interventions.[45] As reported by Ipkeazu et al., mapping was integrated into the intervention planning process at state level in Nigeria to prioritise resources to areas with high sex work ‘density’ to achieve high coverage of basic intervention packages.[15]

Among direct interventions, condom use reduces STI exposure and incidence, and STI treatment acts on existing prevalent infections. By shortening the duration of infectiousness, chains of infection are interrupted, HIV cofactors removed and risk of complications to individuals reduced. Antiretroviral treatment (ART) also greatly benefits those already infected and reduces the likelihood of onward transmission. Morales-Miranda et al. used routinely reported programme data in Guatemala to document scale-up and increasing clinic attendance, examine issues influencing programme retention, and monitor outcomes.[46] An assessment of the Avahan India AIDS Initiative, one of the world’s largest-scale HIV prevention efforts, by Panovska-Griffiths et al., examines the impact of extending coverage to more sex workers.[10] Cost-effectiveness was estimated to be low during programme start-up but improved dramatically as scale increased. At optimal intervention intensity and scale, Avahan could have achieved similar impact with 6% less budget but only 9% of the impact with half the budget.

### Mobilising community

Structural interventions change conditions in sex work settings to reduce vulnerability, support condom use and improve sex workers’ access to services and participation in interventions, both prevention and treatment. In brothels and other venues where sex is sold, condom use policies and ‘house rules’ that shift responsibility for enforcing condom use from individual sex workers to the establishment have overcome inherent power imbalances between sex workers and their clients, and led to rapid increases in condom use and decreased HIV/STI transmission.[5,6]

Sex worker mobilisation and empowerment address similar power differentials. Through solidarity and collective action—beginning with participation in peer interventions—sex workers in different settings have changed not only the ground rules concerning condom use but other underlying conditions that affect their work and lives.[6,28–30,47] Other examples of structural change include working with police on ‘enabling’ environments, preventing violence and raising legal literacy for sex workers, improving access to condoms and clinical services through social marketing and ‘satellite’ clinics in sex work areas, and training clinic personnel to provide high-quality, non-judgmental services.[23]

Such structural interventions have been prominent in Asian responses to HIV.[6,25,26,28] The two main approaches–*top down*, such as regulations on condom use in sex venues, and *bottom up*, by mobilising sex worker communities as active partners in prevention—are potentially complementary. [6,48,49] Recent systematic reviews of sex worker interventions in Africa found few examples of structural interventions and little attention to sex worker mobilisation or empowerment.[13,49] Donor policies that restrict funding to a narrow range of services may result in poor linkages between outreach efforts and facility-based services, low uptake and limited community participation.

Vassall et al. report on a cost-effectiveness analysis conducted in two Avahan districts.[11] A two-stage process related epidemic outcomes to community mobilisation, and costs of interventions to outcomes. Estimated costs per DALY were found to be low for India and actually cost-saving when averted costs of ART were factored in. Wirtz et al. estimate added benefits of community empowerment—assumed to result in 50% less inconsistent condom by sex workers—and equitable ART access for sex workers.[50]

Early sex worker community mobilisation efforts developed in a step-by-step empirical manner.[25,28,29,47] Sadhu et al. compare a capacity building scheme involving mentor communities to more common NGO-guided approaches.[51] Tenni et al. explore overlapping areas of community mobilisation, violence prevention, and police advocacy. They present evidence of negative policing practices towards sex workers—including violence, extortion of money and sex, police crackdowns, confiscation of condoms and arrests—which increase sex workers’ vulnerability and hinder HIV prevention work.[52] Examples from Australia, Thailand, Papua New Guinea, India, Nepal, Kenya and Ghana demonstrate that collaboration with police can increase sensitisation and communication, improve sex worker safety and increase access to services.

### Providing treatment

Due to their generally much higher HIV burden, sex workers are among those most in need of antiretroviral therapy (ART).[2] ART also has potential to prevent onward transmission, and the complementarity of basic condom/STI interventions in sex work and ART scale-up in the general population has been described.[17] Given the high potential for transmission in sex work, even more prevention benefit would be expected from reaching high levels of ART coverage among HIV-positive sex workers. Yet a recent systematic review reported few programmes offering HIV testing or ART to sex workers in Africa.[13]

Cianci et al. assessed the incremental cost of providing ART to sex workers.[53] Using data from a sex worker clinic in Burkina Faso, they estimate the mean annual cost of service provision for female sex workers on ART to be only 25% higher than for those not on ART, and conclude that the costs are comparable to those for other population groups in Africa.

Sustaining HIV viral suppression and immunological improvement requires that individuals engage and remain in the HIV care cascade with good adherence. Mountain et al. report encouraging levels of ART initiation, adherence, retention, and treatment response among sex workers in a systematic review and meta-analysis of 27 studies, 17 from Africa.[54] ART coverage among female sex workers was found to be variable, based on limited data from research sites, and suggests that overcoming barriers to clinical service access is critical.

Donastorg et al. developed an integrated programme to promote prevention and care for Dominican female sex workers living with HIV, including individual counseling and education; peer navigation; clinical provider training; and community mobilisation.[55] They report on a baseline assessment of 268 positive sex workers, more than half of whom had detectable viral load. The findings highlight significant barriers to viral load suppression and STI prevention among sex workers living with HIV, and gaps in the continuum of HIV care and treatment.

### The way forward

The articles in this supplement argue for greater policy and programme support for efficient, scaled and targeted interventions with sex workers in order to improve control of HIV and related STI epidemics. At policy level, this includes structural interventions to increase condom use, reduce vulnerability and remove barriers to prevention and treatment. Implementation science shows how to identify areas where sex work takes place, design standardised packages of effective interventions, monitor progress and estimate outcomes—from programme participation to averted infections and retention on treatment. Sex workers should be involved as active participants in improving policies and programmes.

The fundamentals have been clear for decades. Interventions such as peer outreach, condom policies and programming, STI services and ART have long been shown to be feasible and effective, and are remarkably consistent across countries and regions.[12,13,20,21,26,28] Such direct interventions act proximally during sexual contact to reduce probabilities of transmission—condom use (by reducing exposure), STI treatment and ART (by shortening duration of infectiousness, reducing HIV cofactor effects, suppressing viral load). Structural interventions act synergistically, by changing conditions under which sex work takes place—reducing vulnerability, removing barriers and making direct interventions easier to adopt, more frequently applied or more effective.[13,22,23] Peer-based outreach and community mobilisation interventions facilitate both direct and structural interventions and provide a critical link between the sex worker community and health services.

Balance is important. Narrowly conceived programmes with single interventions—to increase condom use, for example, or test sex workers for HIV—run the risk of alienating sex workers and reducing impact. Programmes should thus strive to provide services that sex workers need and value, and to build trust by involving sex workers in decisions about how services are provided, especially those, like HIV testing, which may feel threatening. Experience shows that the more services meet sex workers’ perceived needs, the more likely they will be to utilise them and contribute actively to interventions. A mobilised community that trusts providers is a powerful platform on which to expand services.

Programmes should attempt to reach high coverage of the most active sex workers as most of the effect of interventions on HIV transmission is achieved by reaching those with the highest rates of partner change.[3,17] Determining eligibility based on level of risk is of course impractical and unethical to make on an individual basis—any sex worker who asks for services should receive them. On the other hand, programmes must decide how to allocate resources, beginning with decisions about which areas to target with outreach and peer interventions. High coverage is critical in areas with overt, full-time, high-volume sex work. The more difficult and resource-intensive task of identifying hidden, part-time sex workers—including ‘transactional’ sex—with few clients may be a secondary public health priority.

In summary, targeted treatment and preventions interventions with sex workers should be scaled up first and foremost in order to reduce the high HIV/STI burden and avert serious morbidity and mortality among sex workers themselves. Feasibility has been demonstrated as has the vital importance of such actions in controlling sexual transmission. The imperative for Africa is rapid scale-up of targeted prevention and treatment, linking outreach and facility-based services, and facilitated by policies and action to improve conditions that place sex workers at high risk and stand in the way of service access. The opportunity is a wealth of accumulated experience working with sex workers in diverse settings, in Africa as well as other regions, which can be tapped to make up for lost time. Elsewhere, even in countries with strong interventions and services for sex workers, an emerging challenge will be sustaining them in the face of declining global resources.

**Key Points**

- Sex work is an important driver of sexual transmission regardless of stage or classification of HIV epidemic. Yet, high-incidence transmission between sex workers and their clients can be interrupted.
- The disproportionate burden of HIV borne by sex workers calls for expedited and facilitated access to appropriate services. Targeted prevention and treatment interventions should be scaled up first and foremost to reduce high HIV/STI burden and avert serious morbidity and mortality among sex workers themselves.
- Experience from Asia has demonstrated the feasibility of protecting sex work, averting secondary transmission beyond sex workers and their clients, and, when implemented at sufficient scale, halting established HIV/STI epidemics. Feasibility and effectiveness have also been shown in Africa, but funding for interventions in sex work remains marginal and large-scale responses are rare.
- Direct interventions should include peer-based outreach, condom programming and appropriate clinical services, and should be supported by structural interventions to reduce vulnerability, facilitate condom use and promote participation and ownership by sex workers.
- Community mobilisation promotes sex worker participation and uptake of services, helps to overcome structural barriers and build a sustainable response. Provision of more comprehensive services addresses sex workers’ broader health and social needs and, in doing so, may also increase engagement and participation of sex workers, and cost-effectiveness of the response.
- Sex work takes place in many contexts and takes many forms, from overt and full-time to part-time and ‘transactional’. While any sex worker who seeks services should receive them, programmes should prioritise coverage of overt, high-volume sex work as a first step to interrupting transmission and controlling epidemics.
- Implementation of both direct and structural interventions should be supported by community mapping and monitoring involving sex workers, where confidentiality and data protection are ensured.
- The articles in this supplement argue for greater policy and programme support for efficient, scaled and targeted implementation of interventions with sex workers in order to improve control of HIV and related STI epidemics.
